# Four novel mutations in the *ALPL* gene in Chinese patients with odonto, childhood, and adult hypophosphatasia

**DOI:** 10.1042/BSR20171377

**Published:** 2018-08-29

**Authors:** Lijun Xu, Qianqian Pang, Yan Jiang, Ou Wang, Mei Li, Xiaoping Xing, Weibo Xia

**Affiliations:** Department of Endocrinology, Key Laboratory of Endocrinology, Ministry of Health, Peking Union Medical College Hospital, Chinese Academy of Medical Sciences, Beijing 100730, China

**Keywords:** ALPL gene, HPP, TNSALP

## Abstract

Hypophosphatasia (HPP) is a rare inherited disorder characterized by defective bone and/or dental mineralization, and decreased serum alkaline phosphatase (ALP) activity. *ALPL*, the only gene related with HPP, encodes tissue non-specific ALP (TNSALP). Few studies were carried out in *ALPL* gene mutations in the Chinese population with HPP. The purpose of the present study is to elucidate the clinical and genetic characteristics of HPP in five unrelated Chinese families and two sporadic patients. Ten clinically diagnosed HPP patients from five unrelated Chinese families and two sporadic patients and fifty healthy controls were genetically investigated. All 12 exons and exon–intron boundaries of the *ALPL* gene were amplified by PCR and directly sequenced. The laboratory and radiological investigations were conducted simultaneously in these HPP ten patients. A 3D model of the TNSALP was used to predict the dominant negative effect of identified missense mutations. Three odonto, three childhood, and four adult types of HPP were clinically diagnosed. Ten mutations were identified in five unrelated Chinese families and two sporadic patients, including eight missense mutations and two frameshift mutations. Of which, four were novel: one frameshift mutation (p.R138Pfsx45); three missense mutations (p.C201R, p.V459A, p.C497S). No identical mutations and any other new *ALPL* mutations were found in unrelated 50 healthy controls. Our study demonstrated that the *ALPL* gene mutations are responsible for HPP in these Chinese families. These findings will be useful for clinicians to improve understanding of this heritable bone disorder.

## Introduction

Hypophosphatasia ((HPP), OMIM: 146300, 241500, 241510) is an inborn error of metabolism characterized by impaired mineralization of bones and teeth, and reduced serum alkaline phosphatase (ALP) activity. It is caused by loss-of-function mutations in the *ALPL* gene (MIM 171760), which is located on chromosome 1p36.1 and consists of 12 exons distributed over 50 kb, encoding tissue non-specific ALP (TNSALP) [1]. TNSALP is a homodimeric enzyme with each monomer composed of 524 amino acids, not only expressed richly in bone, liver, and kidney, but also expressed in the central nervous system, fibroblasts, and endothelial, and other cell types. In the physiological conditions, it functions as an ectophosphatase to hydrolyze inorganic pyrophosphate (PPi) to phosphate (Pi) for the formation of hydroxyapatite, and this balance is essential for the bone mineralization. Thus decline in the activity due to mutations in the *ALPL* gene will lead to various degrees of hypomineralization [2,3].

The diagnosis of HPP is made on the basis of clinical, biochemical, and imaging features. Low serum ALP activity is the biochemical hallmark accompanied by an increased level of pyridoxal-5′-phosphate (PLP). The clinical manifestations of HPP are highly variable, ranging from stillbirth without mineralized bone to early tooth loss without bone symptoms. Based on the age at diagnosis and the severity of HPP, six phenotypes are currently recognized: perinatal (lethal), perinatal (benign), infantile, childhood, adult, and odontohypophosphatasia (Odonto HPP). The prevalence of severe HPP: perinatal (lethal) and infantile forms, has been estimated to be between 1/100000 and 1/300000. The mild forms of HPP (perinatal (benign), childhood, adult and, odonto HPP) are more common than severe forms [4–6]. *ALPL* gene mutation analysis is necessary to document the pattern of inheritance and to understand the recurrence risk of HPP. Up to now, more than 300 distinct *ALPL* mutations have been recognized and associated with various forms of HPP (http://www.sesep.uvsq.fr/03_hypo_mutations.php). The most prevalence of these reported *ALPL* mutations were missense mutations, accounting for 74.7%. The remaining mutations were small deletions (10.9%), splicing mutations (5.6%), nonsense mutations (3.9%), small insertions (2.5%), large deletions (1.4%), complex deletion/insertion (0.7%). The great numbers of *ALPL* missense mutations lead to the strong allelic heterogeneity, which was believed to be responsible for the high clinical heterogeneity [7]. It is clear that HPP expressivity is highly variable, ranging from neonatal death to stillbirth without mineralized bone to early tooth loss or arthritic problems manifesting without bone symptoms in adult life. Whyte et al. [9]. initially revealed that HPP severity generally reflected the inheritance pattern through a mutant allele dosage effect. Subsequently, they documented that this high clinical heterogeneity was largely explained by inheritance patterns: autosomal recessive compared with autosomal dominant transmission of at least 275 mutations (74% being missense), which revealed that severe forms of HPP is usually recessive inherited, whereas autosomal dominant or autosomal recessive is found in mild HPP. A few mutations in the *ALPL* gene were found consistently in North American, European, and Japanese: the mutation F310L and T1559del were commonly associated with the relatively mild and lethal forms of HPP in Japanese patients [2]; the mutation E191K was the most frequent in Caucasian patients with mild HPP, may be because of a founder effect [8]. However, to date, only three studies were carried out in *ALPL* gene mutations in Chinese population with HPP [10–12].

Though *ALPL* gene mutation analysis is necessary for HPP, Whyte [13] revealed that the diagnosis of HPP is usually established by the combination of low ALP, elevated TNSALP substrates, and typical clinical symptoms, not by genetic confirmation. Especially for adults, Mornet et al. [14] reported that perhaps 1 out of every 300 people was carrying an *ALPL* mutation in Europe. Patients with the childhood form have broad-ranging severity, mostly occurs after the first year of life and presents with short stature, delayed walking, and a waddling gait due to bone deformities. Premature loss of primary teeth is the classical manifestation of the childhood HPP, which contributes to the diagnosis of HPP. The adult HPP frequently occurs during middle age and features as osteomalacia, chondrocalcinosis, osteoarthropathy, and stress fractures. Some patients characterize with premature loss of permanent teeth. Patients with odonto HPP usually present with dental complications at any time without radiographic or histopathologic evidence of rickets or osteomalacia [13,15].

In our report, we analyzed the *ALPL* gene in five unrelated families and two sporadic patients with childhood, odonto and adult HPP, and described the mutation types, clinical characteristics, laboratory and radiographic findings, to investigate the phenotype–genotype correlations for improving our understanding of this heritable bone disorder.

## Materials and methods

### Subjects

Five unrelated Chinese families and two sporadic patients comprising ten individuals were studied in the Department of Endocrinology, Peking Union Medical College Hospital (PUMCH). Their parents were non-consanguineous, and all the subjects were from Han ethnic group. Clinical manifestations, physical examinations, laboratory results, and radiology results were investigated. Clinical diagnosis of HPP depended on clinical manifestation and the decreased total ALP activities. The present study was approved by the Ethics Committee of PUMCH.

### Biochemical analysis

Fasted blood samples were collected and placed at room temperature for 30 min, and then centrifuged at 3000 rpm for 10 min to separate the serum for analysis. Twenty-four-hour urine calcium and the blood biochemical parameters including serum calcium (Ca), serum phosphate (P), serum total ALP were measured spectrophotometrically using routine assays in the central laboratory of PUMCH. Serum intact parathyroid hormone (iPTH) and serum 25-hydroxyvitamin D (25(OH)D) were analyzed by an automated Roche electrochemiluminescence system (E170 Roche Diagnostics, Basel, Switzerland). Serum 1,25-dihydroxyvitamin D [1,25(OH)_2_D] level was determined with 1,25(OH)_2_D ^125^I RIA Kit (Diasorin Inc., Stillwater, MN, U.S.A.).

### Radiography and bone mineral density

Radiography studies were performed at the Department of Radiology of PUMCH. The X-ray of thoracic and lumbar vertebrae, femur, and pelvis were measured to detect abnormalities. Bone scan was performed using technetium-99m-MDP (Infinia Hawkeye, GE, U.S.A.) according to the standard protocols.

The bone mineral density (BMD) of the lumbar spine vertebrae 1–4 (L1–L4) and the right proximal femur, including the femoral neck and total hip were measured using dual energy X-ray absorptiometry densitometer (DXA, GE Lunar, U.S.A.) at Department of Radiology at the PUMCH. Height and weight of the subjects were measured using standardized equipment.

### Sequencing analysis of *ALPL*

Genomic DNA of the probands and their relatives available were extracted from peripheral blood leukocytes using the QIAamp DNA Blood Kit (Qiagen, Germany). All 12 exons and intron–exon boundaries of *ALPL* were amplified by PCR primers designed using the Oligo 7 Primer Analysis software (Supplementary Table S1). Taq DNA polymerase (Takara, Japan) and its standard buffer were used in all reactions under the following conditions: initial denaturation at 95° for 5 min, followed by 35 cycles at 94°C for 30 s, 50–60°C for 30 s, and 72°C for 1 min. The resulting PCR products were directly sequenced using an automated ABI 3730XL sequencer according to the manufacturer’s protocol. Sequence alignment was performed using the Basic Local Alignment Search Tool (BLAST) on the National Center for Biotechnology Information database. The identified *ALPL* mutations were subsequently investigated in their relatives using the same method, and also analyzed in 50 unrelated Chinese Han subjects, who were the volunteers for the epidemiological investigation of osteoporosis throughout the country.

### Bioinformatics analysis of mutations

The bioinformatics tools PolyPhen-2 (http://genetics.bwh.harvard.edu/pph), SIFT (http://sift.jcvi.org/), and Mutation Assessor software (http://mutationassessor.org/r3/) were used to predict the effects of missense mutations on protein structure and function. Multiple sequence alignments of TNSALP protein in vertebrate species were generated by using UCSC Genome Bioinformatiocs database (http://genome.ucsc.edu/). A 3D model of the TNSALP (PDB ID: 1EW2), which has been previously constructed [16], was used to predict the dominant negative effect of mutations. Mutation-related residues in the present study were positioned using the open source PyMOL software.

## Results

### Clinical features of the subjects

The clinical findings of the five families and two sporadic patients, all together comprising ten patients with HPP were shown in Table 1.

**Table 1:** Clinical characteristics of the patients and their families

	FM1-1	FM1-2	FM2-1	FM2-2	FM3-1	FM4-1	FM4-2	FM5-1	PA-6	PA-7
Phenotype	Odonto	Odonto	Childhood	adult	childhood	odonto	adult	childhood	adult	adult
Age of onset (ys)	1	1	8 mons	31	1	1	NA	7	33	53
Gender (F/M)	F	M	F	F	M	M	M	M	M	F
Height (cm)	111.4 (6 y) (≤1SD)	NA	118 (8 y) (≤3SD)	NA	160.5 (15 y) (≤2SD)	NA	NA	166 (43 y) (≤1SD)	161 (39 y) (≤2SD)	150 (57 y) (≤2SD)
Serum ALP (U/l)	22 (58–400)	29 (58–400)	6 (30–120)	28 (30–120)	26 (58–400)	17 (58–400)	29 (30–120)	negative	23 (30–120)	13 (30–120)
**BMD**										
L2–L4 (g/cm^2^) (T/Z score)	NA	NA	0.504 (0.6)^*^	NA	0.500 (−1.5)^*^	NA	NA	0.711 (−3.2)^†^	0.911 (−1.6)^†^	0.961 (−1.5)^†^
Femoral neck (g/cm^2^) (T/Z score)	NA	NA	0.560 (0.63)^*^	NA	0.401 (−3.74)^*^	NA	NA	0.427 (−4.2)^†^	0.570 (−3.1)^†^	0.763 (−1.4)^†^
Total hip (g/cm^2^) (T/Z score)	NA	NA	0.506 (−0.15)^*^	NA	0.363 (−4.53)^*^	NA	NA	0.401 (−4.5)^†^	0.714 (−2.1)^†^	0.928 (−0.4)^†^
**Clinical characteristics**										
Early deciduous tooth loss	Yes	Yes	Yes	No but sparse teeth	Yes	Yes	No but sparse teeth	Yes	No	No
History of fracture	No	No	No	No	Yes (once)	No	No	Yes (three times)	No	No
Severity of rickets/ osteomalacia	No	No	Moderate	No	Severe	No	No	Extremely severe	No	No
Bone deformity	No	No	Yes (rachitic chest)	No	Yes (rachitic rosary, enlargement of wrists, scoliosis, subluxation of the bilateral hip)	No	No	Yes (rachitic chest, valgum deformity of right genu)	No	No
Calcium pyrophosphate dihydrate deposition disease (CPPD)	No	No	No	No	Yes	No	No	Yes (stiffness of the left knee-joint)	Yes (pain in arthrosis; calcific periarthritis)	Yes (pain in left shoulder, wrists and elbows)
Skeletal hyperostosis	No	No	No	Yes (slight skeletal hyperostosis in both knee joints)	No	Yes (cortical thickening in fibula and tibia bone)	Yes (cervical bone hyperostosis)	No	Yes (vertebral hyperotosis)	Yes (hyperostosis at thoracic and lumbar vertebra)

The patient from family was indicated in FM; the sporadic patient was indicated in PA. Abbreviation: NA, not available. ys, years. mons, months

^*^, The Z scores at L_1_–L_4_, femoral neck and total hip of young patients were calculated by comparison with the age-specific BMD reference value of Chinese children and adolescents.

^†^, The T scores at L_1_–L_4_, femoral neck and total hip of adult patients were calculated by comparison with the age- and sex-match adult.

The female to male ratio was 4:6. There were three childhood forms (FM2-1, FM3-1, M5-1), three adult forms (FM2-2, PA-6, PA-7), one suspected adult form (FM4-2), and three odonto forms (FM1-1, FM1-2, FM4-1) in the present study. Early deciduous tooth loss was observed in all the HPP patients of childhood and odonto forms. Sparse teeth without early tooth loss was found in two adult HPP patients (FM2-2 and FM4-2). Fragility fracture occurred in FM3-1 and FM5-1. Frequent fractures and poor fracture healing was found in FM5-1. Muscle weakness occurred in FM3-1. Rickets-like changes were observed in all the HPP patients of childhood forms (FM2-1, FM3-1, and FM5-1), including short stature, waddling gait, pectus excavatum, and bowed legs. Calcium pyrophosphate dihydrate deposition disease (CPPD) was observed in four patients, of which two were childhood forms (FM3-1 and FM5-1) and two were adult forms (PA-6 and PA-7). Skeletal hyperostosis was found in five patients (FM2-2, FM4-1, FM4-2, PA-6, PA-7).

**Figure 1:**
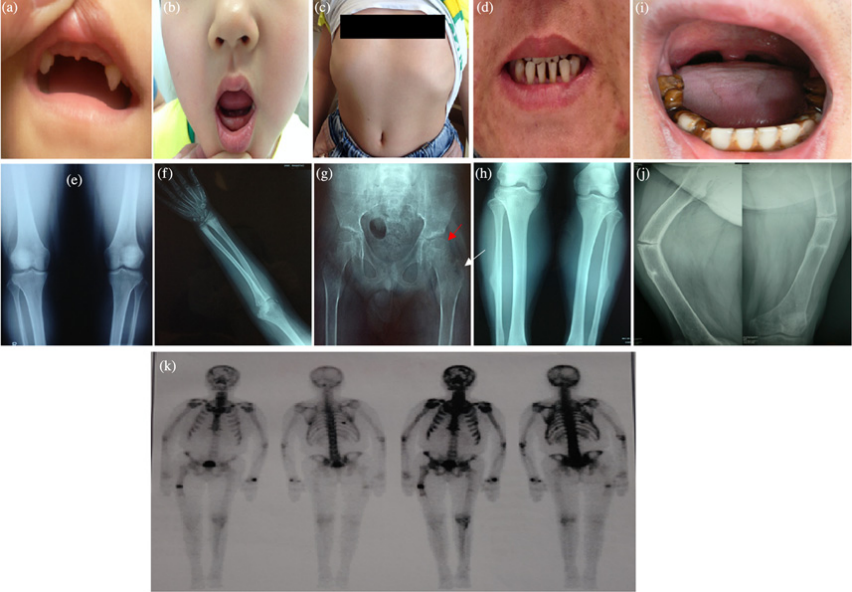
The clinic characteristics and radiographic signatures of the HPP patients. (**a**,**b**) Early deciduous teeth loss of FM1-1 and FM2-1. (a) FM1-1 had only two maxillary canines left at 6 years of age; (b) FM2-1 had only eight primary teeth left at 8 years of age. The permanent teeth present were all molars. (**c**) Pectus excavatum of FM2-1; (**d**) sparse teeth of FM2-2; (**e**) radiographs of both knees of FM2-2 showed slight skeletal hyperostosis; (**f**) radiographic examination of FM3-1 showed signs of rickets in the distal ulna and radius; (**g**) the anteroposterior of the pelvis of FM3-1 showed subluxation of the bilateral hip (red arrow) and calcium deposition adjacent to the great trochanter of the left femur (white arrow); (**h**) X-ray examination of FM4-1demonstrated cortical thickening in fibula and tibia bone; (**i**) early deciduous teeth loss of FM5-1. The permanent teeth present were all lower incisors and lower molars, and the lower molars displayed hypocalcified enamel; (**j**) fracture lines of humerus and femur of FM5-1; (**k**) bone scan of FM5-1 showed multiple areas of increased tracer uptake in the skull, ribs, and femurs.

Proband 1 (FM1-1) was a 6-year-old girl who presented with early deciduous teeth loss at the age of one. She was the first child of non-consanguineous healthy parents. Her birth weight was 3500 g. She began to spontaneously lost teeth at 1 year of age, and she had only two primary teeth left at 6 years of age. The permanent teeth present were two maxillary canines (Figure 1a). During childhood the patient suffered from respiratory infections twice, but never needed any ventilatory treatment. On physical examination, her height was 111.4 cm (−1SD), and weight was 19 kg. She had no history of fractures, bone pain, delay in walking, or waddling gait. Laboratory tests showed low serum ALP activity (22 U/l, normal range for children was 58–400 U/l). Serum Ca was normal while the serum P was slightly high (2.03 mmol/l, normal range for children was 1.29–1.94 mmol/l). Serum 1,25(OH)_2_D (17.4 pg/ml, normal range was 19.6–54.3 pg/ml), and iPTH (8 pg/ml, normal range was 15–65 pg/ml) were both reduced. Her radiological examination and kidney ultrasound were normal. The proband presented with dental complications without clinical or radiographic evidence of rickets, thus she was clinically diagnosed with odonto HPP.

FM1-2, the FM1-1’s younger brother, a 2-year-old boy, who also presented with early deciduous teeth loss since 1 year old. The physical examination was unremarkable. Similar to his elder sister, he had no history of fractures, bone pain, or any bone and joint deformity. Laboratory results showed low serum ALP activity (29 U/l, normal range for children was 58–400 U/l). Serum Ca (2.77 mmol/l, normal range was 2.13–2.70 mmol/l), and P (2.24 mmol/l, normal range for children was 1.29–1.94 mmol/l) were both high. Serum iPTH (16.6 pg/ml, normal range was 15–65 pg/ml) was low but in the normal range. The proband only showed typical dental complications, she therefore was diagnosed with odonto HPP.

Their parents had no history of early deciduous tooth loss and bone fracture. Serum ALP levels were 33 U/l and 48 U/l (30–120 U/l) in their father and mother, respectively.

Proband 2 (FM2-1) was an 8-year-old girl presenting with early deciduous teeth loss, difficulty of teeth eruption, weakness in lower limbs, and bone pain after long walking. She was delivered at term through caesarean section with a birth weight of 4800 g. She began to spontaneously lose teeth at 8 months of age, and she had only eight primary teeth left at 8 years of age. The permanent teeth present were all molars (Figure 1b). On physical examination, her height was 118 cm (−2SD), and weight was 21 kg (−1SD). The proband exhibited rickets-like changes with waddling gait and pectus excavatum (Figure 1c). Sometimes she complained about bone pain in hip. She had no history of fractures. Laboratory tests showed low serum ALP activity (6 U/l, the normal range for children was 58–400 U/l). Serum Ca (2.68 mmol/l, normal range was 2.13–2.70 mmol/l) and P (1.96 mmol/l, the normal range for children was 1.29–1.94 mmol/l) were slightly high but in the normal range. Serum levels of iPTH was decreased (12.6 pg/ml, the normal range was 15–65 pg/ml). She was clinically diagnosed with childhood HPP.

FM2-2, FM2-1’s mother, a 31-year-old woman, did not show any clinical symptoms related to HPP except for sparse teeth (Figure 1d) and slight bone pain in knee joints. She had no history of fractures. Biochemical tests showed ALP activity was slightly decreased (28 U/l, the normal range for adults was 30–120 U/l). Serum levels of Ca, P, and iPTH were all in the normal range (Table 2). X-ray showed slight skeletal hyperostosis in both knee joints (Figure 1e). Thus, she was clinically diagnosed with suspected adult HPP.

**Table 2:** Laboratory findings of the six families and three patients with HPP

	FM1-1	FM1-2	FM2-1	FM2-2	FM3-1	FM4-1	FM4-2	FM5-1	PA-6	PA-7	Reference value
Serum calcium (mmol/l)	2.45	**2.77**	2.68	2.38	2.48	2.52	NA	2.33	2.14	2.48	2.13–2.70
Serum phosphate (mmol/l)	2.03^*^	2.24^*^	1.96^*^	1.26^†^	1.89^*^	1.82^*^	NA	1.43^†^	1.11^†^	1.19^†^	1.29–1.94^*^
											0.81–1.45^†^
Serum ALP (U/l)	22^*^	29^*^	6^*^	**28**^†^	26^*^	17^*^	**29**^†^	**Negative^^‡^^**	**23**^†^	**13**^†^	58–400^*^
											30–120^†^
Serum 25 (OH)D (ng/ml)	NA	NA	30.7	NA	10	17.3	NA	23.3	15.9	13.7	8–50
Serum 1,25(OH)_2_D (pg/ml)	**17.4**	NA	34.29	NA	NA	NA	NA	47.37	53.87	NA	19.6–54.3
Serum iPTH (pg/ml)	**8**	16.6	**12.6**	49.2	**8**	54	NA	31.4	31.9	43.4	15–65
24-h urine calcium (mmol)	0.41	NA	1.60	NA	4.64	2.78	NA	3.29	6.53	3.39	2.5–7.5
Nephrocalcinosis	no	NA	no	NA	no	no	NA	no	yes	no	

Abnormal results were indicated in bold. Abbreviation: NA, not available.

^*^, Indicated the normal range for serum phosphate, serum ALP in patients from 2 to 18 years old are 1.29–1.94 mmol/l, 58–400 U/l. ^†^, Indicated the normal range for serum phosphate, serum ALP in adult patient are 0.81–1.45, 30–120 U/l .

^‡^, Phosphatase value was negative in FM5-1 after repeated measurement.

It was worthy to note some interesting clinical features found in Proband 3 from FM3. FM3-1, a 15-year-old boy, was referred to our clinic for his bone pain and weakness of lower extremities. He was the second child of non-consanguineous healthy parents. He was delivered at term through vaginal delivery after an uneventful pregnancy. He had premature loss of deciduous teeth a few months after eruption. During infancy, he developed recurrent respiratory infections, but never needed any ventilatory support. At age of 14 years, he suffered from traumatic bone fracture at lumbar vertebra. The proband started to walk at the age of 1 displaying a waddling gait. Later on, he had mild proximal muscle weakness in his lower limbs and difficulty in walking and inability to climb stairs. He often complained about bone pain in hips after a long walk. Physical examination revealed rickets-like changes with rachitic rosary and bone deformity in lower limbs. His height was 160.5 cm (−2SD), while the target height was 171.0 cm. The tendon reflexes of limbs were slightly decreased. Electromyography (EMG) of the limbs showed low amplitude motor action potential. Muscle biopsy specimens of the left quadriceps were performed by light microscopy and histochemistry, showing ragged red fibers, deeper succinate dehydrogenase (SDH) histochemical staining on vessel wall and type II muscular fibers predominance. Laboratory tests showed the low serum ALP activity (26 U/l, the normal range for children was 58–400 U/l). Serum Ca and P were in the normal range. Serum levels of iPTH was decreased (8 pg/ml, the normal range was 15–65 pg/ml). Serum levels of high sensitive C-reactive protein (hsCRP) was increased significantly (10 mg/l, the normal range was 0–3 mg/l). The muscle enzymes were normal. He had low BMD by DXA (Table 1). Radiographic examination showed signs of rickets in the distal ulna and radius and subluxation of the bilateral hip (Figure 1f,g). Moreover, the pelvis X-ray showed calcium deposition adjacent to the great trochanter of the left femur (Figure 1g). Clinical findings, laboratory results, and radiological features were consistent with childhood HPP, thus the proband was diagnosed with childhood HPP. His mother had moderate decayed teeth, but she had no teeth loss and any clinical symptoms related to HPP. Serum ALP levels were slightly low (29 U/l, the normal range for adult was 30–120 U/l).

Proband 4 (FM4-1) was a 16-year-old boy with early deciduous teeth loss. He was the first child of non-consanguineous parents. He was normal spontaneous full-term delivery with the weight of 4000 g. He had premature loss of deciduous teeth a few months after eruption. At age of 16 years, he had lost three teeth. The permanent teeth present displayed hypocalcified enamel. He had no history of fractures, bone pain, delay in walking, or waddling gait. X-ray showed cortical thickening in fibula and tibia bone (Figure 1h). Serum ALP level was 17 U/l (58–400 U/l). Serum Ca, P, and iPTH were all in the normal range (Table 2). The proband only presented with dental complications, thus he was diagnosed with odonto HPP. The proband’s father (FM4-2) suffered from lumbar spondylolisthesis, cervical bone hyperostosis, and sparse teeth. Serum ALP level was 29 U/l (30–120 U/l). Unfortunately, imaging was not available at this time. In consideration of clinical features and low ALP activity, he was diagnosed with suspected adult HPP. The proband’s mother was asymptomatic.

Proband 5 (FM5-1) was a 43-year-old man with early deciduous teeth loss from 7 years old. At 43 years of age, he had only eight teeth left. The permanent teeth present were all lower incisors and lower molars. The lower molars displayed hypocalcified enamel (Figure 1i). He suffered from frequent fractures since he was 7 years old: the first time of fracture happened in 1980 at the humerus, the second time of fracture happened in 2001 at his left femoral shaft (Figure 1j), and the last time of fracture happened in 2010 at his right femoral shaft (Figure 1j). Physical examination showed short stature, barrel chest, bowed legs, and limited activity in left knee joint. Dual energy ray absorptiometry showed T score of less than −2 (Table 1). Bone scan showed multiple areas of increased tracer uptake in the skull, ribs, and femurs (Figure 1k). Serum ALP level was negative after repeated measurements. Serum Ca, P, iPTH, and 25(OH)D were all in the normal range (Table 2). Clinical features, laboratory results, and radiological findings were consistent with childhood HPP, the proband therefore was diagnosed with childhood HPP. The proband’s older brother had early deciduous teeth loss, recurrent bone fractures from a child, severe deformities at lower extremities, and shortened stature. His father had teeth loss since he was 40 years old, but no history of bone disorders. Unfortunately, blood samples of his brother and parents were not obtained for biochemical measurements and gene testing.

Sporadic patient 6 (PA-6) was a 39-year-old man who presented with recurrent episodes of bone pain in waist and shoulder from 35 years old. He had no history of premature loss of deciduous teeth and bone fractures. At the age of 37 years, he suffered from nephrolithiasis. The physical examination was normal. DXA showed T score of less than −2 and diagnosed osteoporosis (Table 1). Radiographic examination showed calcification at anterior longitudinal ligament, vertebral hyperotosis and, calcific periarthritis. Serum ALP level was 23 U/l (30–120 U/l). Serum Ca, P and, iPTH were all in the normal range (Table 2). His parents had early deciduous teeth loss and were clinically suspected of osteoporosis. Unfortunately, blood samples of his parents were not available.

Sporadic patient 7 (PA-7) was a 57-year-old woman with osteoporosis. At 53 years of age, she recurrently complained about her bone pain and received bisphosphonates therapy. After discontinuance of the drug, serum ALP level was consistently subnormal, serum Ca, P, and iPTH were all in the normal range (Table 2), therefore HPP was suspicious. Radiographic examination showed hyperostosis at thoracic and lumbar vertebra. Her family members were asymptomatic.

### Biochemical parameters

Biochemical parameters in patients with HPP from ten patients were shown in Table 2. All the ten patients showed decreased ALP activities. Besides, in consideration of age influence, serum levels of phosphate were increased in FM1-1, FM1-2, and FM2-1. Serum iPTH was decreased in three patients (FM1-1, FM2-1, and FM3-1). Unfortunately, the best marker of HPP (PLP), was not detected in the present study, since we did not obtain the plasma from the patients, and the remaining serum was not enough to perform PLP examination.

### Mutational of the *ALPL* gene

**Figure 2:**
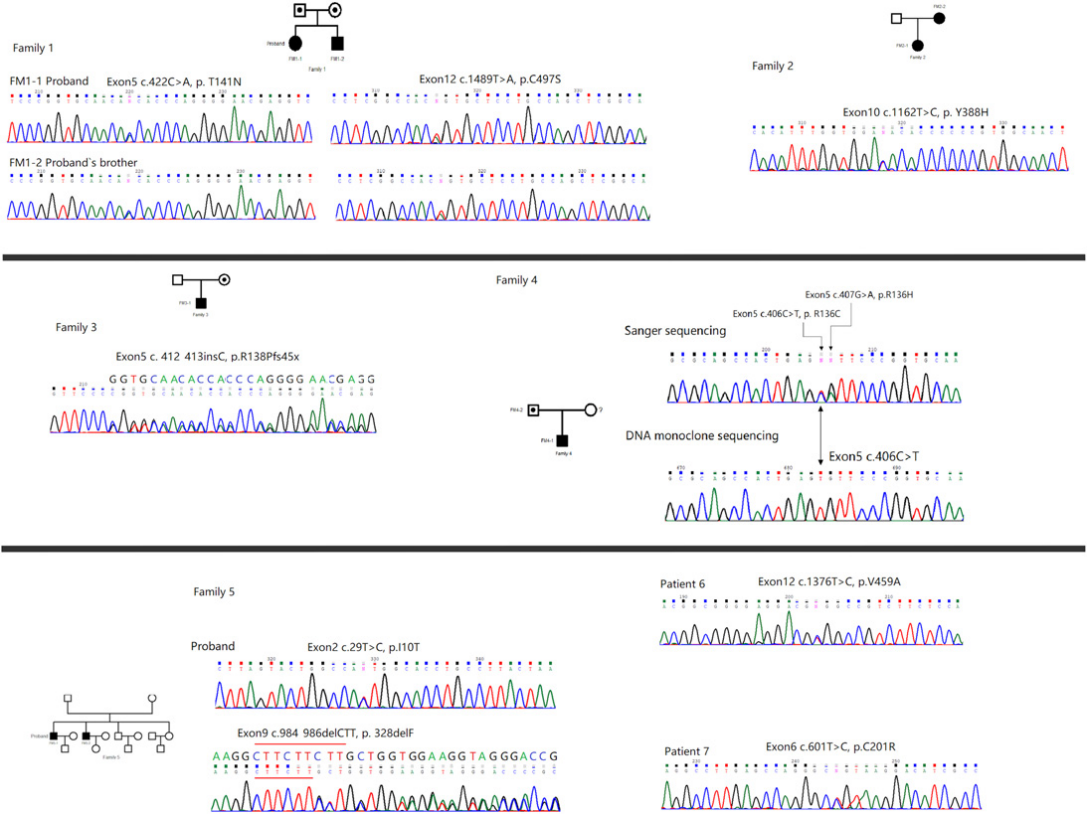
Genetic analysis of *ALPL* in the probands and their relatives. Black symbols represent the affected individuals, open symbols with a point represent the carriers, and open symbols represent the unaffected individuals. Circles and squares indicate the females and males, respectively.

Mutational analysis of the *ALPL* gene was performed (Table 3). Pedigrees and genetic analysis of *ALPL* gene in the probands were shown in Figure 2. Ten mutations were identified in these five families and two sporadic patients. According to Human Gene Mutation Datebase (HGMD) (http://www.hgmd.org/), six mutations in FM1, FM2, FM4, and FM5 were previously identified (p.T141N, p.Y388H, p.R136H, p. R136C, p.I10T, p.328delF); the remaining four mutations were novel: one frameshift mutation c.412_413dupC (p.R138Pfsx45*) was found in FM3; and three missense mutations (p.C497S, p.V459A, p.C201R) were found in FM1, PA-6, and PA-7, respectively. These missense mutations were almost predicted *in silico* to be damaging (Table 3). The mutation I10T was predicted benign with all the three functional predictor softwares (Polyphen2, SIFT, and Mutation Assessor softwares). No identical mutations and any other new *ALPL* mutations were found in the unrelated 50 healthy controls.

**Table 3:** Mutations in the *ALPL* gene found in the six families and three patients

Number	Relationship	Amino acid change	Status	Location of mutated amino acids in 3D model	Function Predict (Polyphen2, SIFT, Mutation Assessor)	Reference
**FM1**						
FM1-1	Proband	T141N	Compound heterozygous	Active site	Damaging	[8]
		C497S^*^		Not shown	Damaging	The present study
FM1-2	Proband’s brother	T141N	Compound heterozygous	Active site	Damaging	[8]
		C497S^*^		Not shown	Damaging	The present study
FM1-3	Proband’s mother	T141N	Heterozygous	Active site	Damaging	
FM1-4	Proband’s father	C497S^*^	Heterozygous	Not shown	Damaging	The present study
**FM2**						
FM2-1	Proband	Y388H	Heterozygous	Crown domain	Damaging	[12]
FM2-2	Proband’s mother	Y388H	Heterozygous	Crown domain	Damaging	12
**FM3**						
FM3-1	Proband	R138Pfs45x^*^	Frameshift	-	-	The present study
**FM4**						
FM4-1	Proband	R136H	Compound heterozygous	Active site	Damaging	30
		R136C		Active site	Damaging	Versailles lab, October 2003
FM4-2	Proband’s father	R136H	Heterozygous	Active site	Damaging	30
**FM5**						
FM5-1	Proband	I10T	Compound heterozygous	Not shown	Benign	Versailles lab, June 2017
		328delF		Homodimer interface	-	25
**PA-6**	V459A*	Heterozygous	Homodimer interface	Damaging	The present study	
**PA-7**	C201R*	Heterozygous	-	Damaging	The present study	

Accession number genomic sequence of the *ALPL* gene: Ref Seq NG_008940.1. Function predict by PolyPhen2 software, score ranges from 0 to 1.000, where 0 is benign, and a high positive number is damaging.

*, Novel mutations in *ALPL*.

**Figure 3:**
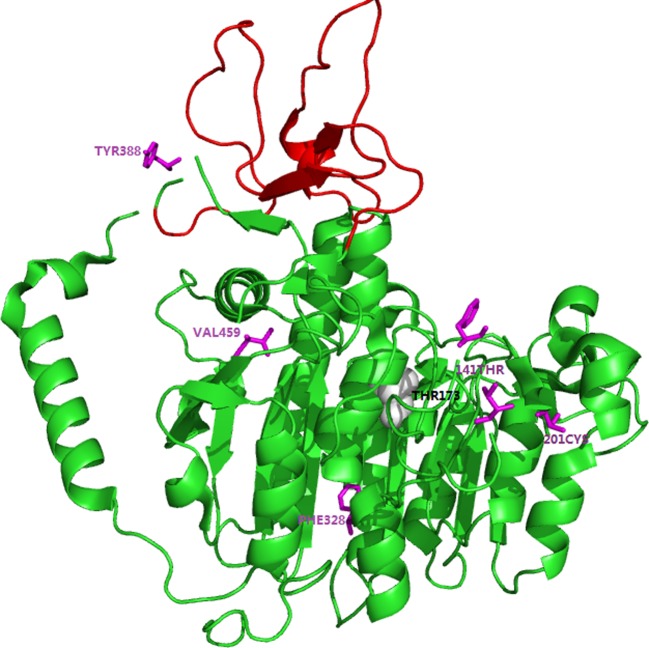
Three dimensional models of TNSALP monomer. The model was based on the crystal structure of the PLAP monomer (PDB ID: 1E2W). The crown domain was indicated by red. The magnesium ion was showed by gray ball. The single heterozygous mutations found in the present study located in the active site (T141), the crown domain (Y388), and the homodimer interface (F328, V459) were highlighted in purple, respectively.

Amongst the three childhood forms of HPP, only FM5-1 was inherited autosomal recessive patterns with compound heterozygous mutation, the other patients with childhood forms were inherited autosomal dominant patterns with heterozygous missense mutation and frameshift mutation (FM2-1 and FM3-1, respectively). FM1 and FM4 were diagnosed with odonto HPP, all of whom were inherited autosomal recessive with compound heterozygous mutations. The remaining four patients with clinical features of adult HPP, were found all inherited autosomal dominant with heterozygous mutations (FM2-2, FM4-2, PA-6, and PA-7). To predict dominant negative effect of these missense mutations affecting residues, we used the 3D modeling of TNSALP (the structure modeling is based on its sequence homology to the placental isozyme, PDB ID: 1EW2) to position and analysis. The results indicated that these residues located particularly in active site (T141N, R136H) area, the crown domain (Y388H), and the homodimer interface (V459A) (Figure 3).

## Discussion

In the present study, totally ten mutations, including four novel mutations and six known mutations, in the *ALPL* gene were identified in these five unrelated Chinese families and two sporadic patients.

TNSALP, as previously reported, is an tissue-nonspecific isozyme and is expressed richly in bone, liver, and kidney, located in the cell surface and functions as an ectophosphatase to hydrolyze monophosphated esters including inorganic PPi and PLP [2,3]. It acts physiologically as a homodimer, there are five domains identified in TNSALP, (i) the active site; (ii) the homodimer interface, which the two monomers related by a two-fold crystallographic axis and crucial for stability and enzymatic function [17]; (iii) the crown domain; (iv) N-terminal α-helix. Hoylaerts et al. [18] observed that both the crown domain and the N-terminal α-helix help stabilize the dimeric structure and determine allosteric properties; (v) the metal-binding site, including two zinc, one magnesium, and one Ca^2+^-binding sites. Three metal-binding sites (two zinc atoms and one magnesium ion) are essential for TNSALP enzymatic activity, whereas the Ca^2+^-binding site does not influence TNSALP catalytic activity [19]. Thus, *ALPL* mutations alter residues at these sites can cause HPP.

Clinical symptoms of HPP varied remarkably, ranging from a perinatal lethal form to odonto HPP. This high clinical heterogeneity is mainly considered as due to the great number of *ALPL* missense mutations [2,15]. The two mild forms of HPP: childhood and adult manner, are known to be inherited in an autosomal recessive (compound heterozygous mutations) or autosomal dominant (single heterozygous mutation). Whereas previous study reported that a large part of mild HPP is due to single heterozygous mutation with a dominant negative effect, which may be responsible for the inhibition of the activity of the wild-type monomer by the mutated monomer in heterodimers. While in the same study, Fauvert et al. [20] found that the residues located in particular region, especially the active site, crown domain, and the homodimer interface were predicted to have dominant effect. In the present study, after locating the identified mutations in 3D structure of the TNSALP, whose crystallographic co-ordinates have been determined by Mornet et al. [19] based on human placental ALP, we found that the single heterozygous mutations R136H, Y388H, and V459A were located in the active site, the crown domain, and the homodimer interface, respectively (Figure 3). C201 is one of the cysteine residues (C139–C201, C489–C497), which is essential for proper folding of monomeric TNSALP [21]. Mornet et al. [19] revealed that cysteine residues at position 201 covalently link to 139 to form disulphide bond (C-139–C-201). Subsequently, Satou et al. [22] demonstrated that the C-139–C-201 disulphide bond helped assist monomeric TNSALP to fold into a tertiary structure, which could be competent to assemble with the counterpart subunit non-covalently. Furthermore, Satou et al. [22] also found that the mutation C201Y had a weak ALP activity, which indirectly suggested the correct disulphide bond in the molecule play crucial role in the dominant effect. Therefore, we suggested that the heterozygous mutations should be responsible for mild odonto, childhood, and adult HPP disorders in these families and sporadic patients.

Odonto HPP, the mildest form of HPP, features dental complications at any age without radiographic or histopathologic evidence of rickets or osteomalacia [13]. FM1 and FM4 were diagnosed with odonto HPP, all of whom inherited autosomal recessive with compound heterozygous mutations. FM1-1 and FM1-2 were siblings and both only presented with early deciduous teeth loss, severe clinical features such as rickets, muscle weakness, and multifocal osteomyelitis were not found. FM4-1 also showed cortical thickening in fibula and tibia bone, which only frequently occurred in adult HPP.

Childhood HPP is probably the most heterogeneous clinical form of HPP, has broad-ranging severity. It is characterized by classical premature loss of deciduous teeth (beginning with incisors). Rickets sometimes causes short stature and skeletal deformities. Bone pain, joint pain (condrocalcinosis and osteoarthritis develop with ageing), low BMD, and some degree of motor impairment and fatigue easily also occurred in some childhood HPP [17,23]. In the present study, all three childhood HPP featured early deciduous teeth loss. Beertsen et al. revealed that premature loss of deciduous teeth was related to insufficient mineralized cementum covering the tooth roots [24].

Amongst the three childhood HPP, as mentioned above, only FM5-1 was inherited autosomal recessive patterns with compound heterozygous mutation, the other two (FM2-1 and FM3-1) carried with single heterozygous mutation: one missense mutation and one frameshift mutation, respectively.

FM5-1 was different from the three childhood HPP mentioned above, except experienced shared early deciduous teeth loss, the patient also suffered from severe rickets, fragility fracture and, hypomineralization. Moreover, the ALP level was extremely low and was negative after repeated measurements, suggesting the patient was severe. Mutational analysis revealed that FM1-1, FM1-2, and FM4-1 carried with compound heterozygous missense mutations, and the analysis of the 3D structural model showed that T141 and R136 were both located at the active site, indicating indirectly affect the function of TNSALP, which may be predicted to be dominant negative effect, suggesting the patients with moderate childhood HPP. FM5-1 carried with heterozygous mutations c.984_986delCTT (p.328delF) and c.29T>C (p.I10T). The mutation I10T was predicted benign with all of three functional predictor softwares (Polyphen2, SIFT, and Mutation Assessor software). However, the mutation p.I10T was previously reported in an infantile HPP patient with homozygous *ALPL* mutation (Versailles lab, June 2017), we therefore considered this mutation as damaging despite of *in silico* prediction. In addition, the 328delF was previously reported in a 12-h year male infant with severe lethal perinatal HPP [25]; 3D structural analysis revealed that F328 was located in the β-sheets and contributed to the stability of the hydrophobic region, indicating deletion of F328 may influence the stability of the hydrophobic region. Michigami et al. [2] demonstrated that 328delF resulted in less than 20% of enzymatic activities of mutant TNSALP proteins. Thus, the results confirmed the severe clinical features of childhood HPP.

The other two childhood HPP carried with single heterozygous mutation. FM2-1 and her mother (FM2-2) both possessed the same mutation Y388H in *ALPL*. FM2-1 manifested premature loss of deciduous teeth and funnel chest, confirming the diagnosis of childhood HPP. However, her mother only featured mild joint pain in knees occasionally and did not present premature loss of deciduous teeth. We therefore suggested that FM2-2 was affected with adult HPP. The same symptoms were observed within the other families, such as FM3. In FM3, FM3-1 carried with a novel frameshift mutation, resulting in a premature stop codon and truncated amino acids. This mutated protein was supposed to be responsible for the severe clinical manifestations of FM3-1, who presented proximal muscle weakness in his lower limbs, waddling gait, mild muscle hypotonia, and signs of rickets. Hypotonia and muscle weakness are well-known features of the infantile forms, few reports have been described in childhood forms. However, his mother, who carried the same mutation, only had moderate decayed teeth, but no teeth loss. These results consistent with previous studies that high phenotypic variability was observed even within the family [26].

Similar to childhood HPP, adult HPP features a wide spectrum of clinical manifestations. The adult form of HPP typically presents poorly healing, metatarsal stress fractures and proximal femoral pseudofractures during the middle age. Some of these patients also suffer from CPPD crystal deposition (chondrocalcinosis or calcific periarthritis) due to the PPi accumulation [17,27]. In this study, all four adult HPP were inherited in an autosomal dominant with single heterozygous mutation and presented with mild symptoms. As reported above, all the mutated acids located at the particular regions of the 3D structure of the TNSALP suggesting a dominant negative effect, confirming the diagnosis of mild adult HPP.

Interestingly, in the present study, PA-6 manifested calcification at anterior longitudinal ligament, which had not been reported before. The exact molecular mechanisms of anterior longitudinal ligament calcification remained unclear. However the pathogenesis of ossification of the posterior longitudinal ligament (OPLL), which was most serious disease in ectopic ossification of spinal ligaments, has been well illustrated [28].

It was worth noting that the PA-7 had only presented with skeletal hyperostosis and low bone density, without presenting with any typical features of HPP, thus the patient was initially diagnosed of osteoporosis and treated with bisphosphonate for long time. After discontinuance of the drug, serum ALP was consistently subnormal, therefore HPP was suspicious. Whereas, bisphosphonates are analogs of PPi that suppress bone turnover but also might deactivate ALP [3,27]. Previous study had reported an adverse effect of the drugs (atypical femoral fractures) that occurred in adult HPP with bisphosphonate treatment [29,30]. Therefore, asymptomatic adult HPP patients diagnosed with osteoporosis were perhaps inclined to develop a typical femoral fractures due to bisphosphonates. Clinicians should be suspicious of HPP when clinical clues included premature loss of deciduous or adult teeth, pseudofractures or recurrent poorly healing metatarsal stress fractures. If HPP was diagnosed, bisphosphonate treatment should be avoided.

There are still some limitations in the present study. First, the number of subjects is not big enough to reveal the phenotype–genotype correlations. Second, PLP, the best markers of HPP, was not detected in the present study since we do not obtain the plasma from the patients, and the remained serum is not enough to perform PLP examination. Lacking the proof of elevated TNSALP substrates, it is not clear, if the patient who does not present with typical clinical and radiographic features of HPP can be really diagnosed with HPP, especially the adult patients. Thus, in the further study we should focus on collecting more clinical data to analyze the relationship and differences amongst the different forms of HPP.

## Conclusion

In conclusion, we described the clinical characteristics and genetic analysis of HPP in five unrelated Chinese families and two sporadic patients. Of which three patients from two families (FM1-1, FM1-2, and FM4-1) were autosomal recessive and were all clinically diagnosed with odonto HPP. One patient from FM5 was autosomal recessive and was clinically diagnosed with childhood HPP. The remaining six patients from three families and two sporadic patients were autosomal dominant and were clinically diagnosed with childhood (FM2-1 and FM3-1) and adult HPP (FM2-2, FM4-2, PA6, and PA-7). Besides, there are total ten mutations were identified including four novel mutations (p.C497S, p.V459A, p.C201R, p.R138Pfsx45) and six known mutations (p.T141N, p.Y388H, p.R136H, p. R136C, p.I10T, p.328delF). To our knowledge, this is the largest number of patients with HPP in the Chinese population. We analyzed the phenotype–genotype correlations in detail, which helps clinicians in improving the understanding of this heritable bone disorder.

## Supporting information

**Table S1.** Primer sequences used to amplify the 12 *ALPL* gene.

## Abbreviations

ALP: alkaline phosphatase
BMD: bone mineral density
Ca: calcium
CPPD: calcium pyrophosphate dihydrate deposition disease
DXA: dual energy X-ray absorptiometry densitometer
EMG: Electromyography
HGMD: human gene mutation database
HPP: hypophosphatasia
iPTH: intact parathyroid hormone
odonto HPP: odontohypophosphatasia
25(OH)D: 25-hydroxyvitamin D
1,25(OH)_2_D: 1,25-dihydroxyvitamin D
OPLL: ossification of the posterior longitudinal ligament
P: phosphate
PLP: pyridoxal-5′-phosphate
PPi: pyrophosphate
PUMCH: Peking Union Medical College Hospital
SDH: succinate dehydrogenase
TNSALP: tissue non-specific ALP
