# Supplementary material for: Four novel mutations in the ALPL gene in Chinese patients with odonto, childhood, and adult hypophosphatasia
Source: Biosci Rep. 2018 Aug 29;38(4):BSR20171377. doi: 10.1042/BSR20171377 (PMC6131208; doi:10.1042/BSR20171377)
Supplement: Supplementary file 1 [file bsr20171377_Supp1.pdf]

Table S1. Primer sequences used to amplify the 12 *ALPL* gene.

Exon	Orientation	Sequence	Length (bp)	AT (℃)
1F	Forward	CACAGAGACAGACGCCAGA	640	63.6
1R	Reverse	CCCTGCCATTAAAGTTCAACC		
2F	Forward	GCAGCATGGAACTCATTGACA	549	55
2R	Reverse	ACTAACACATGCAAGTAGCAGG		
3F	Forward	CAACTATTGCACCCACCTCC	364	56
3R	Reverse	ACACCCTTCCTCCAGAGCC		
4F	Forward	CCCGAGCCTGCCTTGGTA	444	54
4R	Reverse	GCCTGGGCAACAGAGCAA		
5F	Forward	TGGTCAAGGCTATGGGGTCC	467	60
5R	Reverse	CCCCTGTCACGGAAGGAATC		
6F	Forward	GGAAGGGGCTAGAAAAGG	967	57.5
6R	Reverse	CAACCGCAAATCCCCTAATG		
7F	Forward	ATCAGCTCCTCCAATATCCC	487	55
7R	Reverse	ATTTCCGATGCCCTTTCTAC		
8F	Forward	GTGAGGGAAGGAAACAAGTAAAG	351	57
8R	Reverse	GCCTAATTCCAGGAACCAGA		
9F	Forward	CCCAGCCACCATACTCTACCC	383	60
9R	Reverse	CTACAGTGCCACCCGTCTCC		
10F	Forward	GTCAGGTTGAATGGCTGCGTAA	422	60
10R	Reverse	GCTCTGGGCTTGCTGGCTCT		
11F	Forward	GAAGATCCCAGGGGTTACCA	506	56
11R	Reverse	GGTCATTCAGACAACCGTCA		

12F	Forward	ATTCAAGCCAGCCTGGAAG	639	54
12R	Forward	CTAGCGAACGTATTTCTCC		
